# Editorial: Cognitive and mental health improvement under- and post-COVID-19, volume III

**DOI:** 10.3389/fpsyg.2025.1588477

**Published:** 2025-03-19

**Authors:** Yuka Kotozaki, Gabriele Nibbio, Chong Chen

**Affiliations:** ^1^Department of Hygiene and Preventive Medicine, School of Medicine, Iwate Medical University, Morioka, Iwate, Japan; ^2^Department of Clinical and Experimental Sciences, University of Brescia, Brescia, Italy; ^3^Division of Neuropsychiatry, Department of Neuroscience, Yamaguchi University Graduate School of Medicine, Ube, Yamaguchi, Japan

**Keywords:** COVID-19, pandemic, burnout, mental health, social support, urban-rural disparities, suicide rate

This is a part of a collection on the Research Topic of *Cognitive and mental health improvement under- and post-COVID-19*. Please refer to the related Research Topic (Chen et al., 2025; Nibbio et al., 2025).

The COVID-19 pandemic has contributed to a global decline in mental health and cognitive function (Vindegaard and Benros, 2020; Xie et al., 2022; Hampshire et al., 2021; Chen et al., 2023; Galderisi et al., 2024), necessitating effective interventions. While factors such as regular exercise, sufficient sleep, a balanced diet, social connections, engagement with nature, and mindfulness are known to enhance cognition and mental wellbeing (Rebar and Taylor, 2015; Vancampfort and Firth, 2018; Davidson and Dahl, 2018; Chen and Nakagawa, 2019, 2020; Mizumoto et al., 2024; Nibbio et al., 2025), the optimal approaches for specific populations—both healthy and clinical—remain unclear. Further research is required to identify the most effective intervention strategies and elucidate the underlying psychological, physiological, and neurobiological mechanisms through which these interventions exert their benefits.

The aim of this Research Topic is to publish a wide range of studies that help address these unsolved issues and advance our understanding of what activities and interventions help improve cognition and promote mental health. This editorial presents a summary of the main results for the 11 manuscripts.

Zhao et al. aimed to understand job burnout among village doctors during the COVID-19 epidemic and identify influencing factors. A survey of 993 village doctors revealed a burnout incidence of 53.47%, with 54.05% experiencing mild, 33.14% moderate, and 12.81% severe burnout. Key factors associated with burnout included high work pressure, difficulty using WeChat, high practice risk, low economic and technical support, and poor emotional support. The findings highlight the need for better support and resources to alleviate job burnout among village doctors.

Xiao examined the impact of COVID-19 on urban-rural health inequality in China, focusing on the roles of socioeconomic status and social capital. Data from 1,936 participants showed that rural youth had better mental health than urban youth, especially when the pandemic was less severe. Socioeconomic status positively influenced mental health, with a stronger effect for urban youth. Social capital also had a positive effect on mental health, but its impact was similar for both urban and rural populations. The findings highlight the complex relationship between urban-rural disparities, socioeconomic factors, and mental health during the pandemic.

Metzger et al. explored the motivations for healthcare workers and students to volunteer and examined the relationship between volunteering and burnout. Participants (eight healthcare providers, 10 graduate students, 14 undergraduate students) completed a burnout assessment and semi-structured interviews. The results showed that burnout decreased the likelihood of volunteering, but volunteering helped prevent burnout. Most participants volunteered for professional development, to make a difference, or because they felt a sense of responsibility. COVID-19 had a significant impact on both burnout and motivations. The study concluded that while volunteering can help prevent burnout, it may not benefit those already experiencing burnout. Healthcare organizations can promote volunteering by emphasizing its professional development benefits and making it more convenient for students and professionals.

He et al.'s study investigated anxiety and depression in IBD patients during the COVID-19 pandemic and analyzed contributing factors. Among 215 IBD patients, 27% reported anxiety and 34% depression. Factors positively associated with mental health issues included longer waiting times for admission, irregular oral medication, and diet changes. Conversely, timely periodic infusion of biological agents was linked to lower anxiety and depression. Changes in physical activity, sleep, COVID-19 knowledge, and self-prevention measures were not significantly correlated with mental health outcomes. The study emphasizes the importance of maintaining routine treatment and medication, as well as establishing online self-management programs for IBD patients during public health crises.

Rung et al. examined the mental health impacts of the COVID-19 pandemic on Black and White Louisiana residents, focusing on general wellbeing and identifying protective factors like social support, resilience, and social cohesion. The survey revealed that Black individuals experienced higher levels of pandemic-related stress and had lower levels of these protective factors compared to White individuals. However, both groups benefited from social support, resilience, and social cohesion, though these protective effects weakened as pandemic impacts increased. Racial disparities were observed in how these protective factors deteriorated over time. The study highlights the need for targeted interventions to support vulnerable communities, especially minority groups, by enhancing psychosocial resources to mitigate the mental health effects of future crises.

Claessens et al. investigated cognitive and psychological outcomes in non-hospitalized post-COVID-19 patients with persistent symptoms, more than 3 months after infection. A total of 265 patients (61% female, average age 51.7 years) were assessed for anxiety, depression, PTSD, and cognitive symptoms. Results showed that 40% had high anxiety, 43% had depression, 31% had PTSD, and 79% reported cognitive symptoms. Bivariate analysis indicated that a history of psychiatric conditions and physical symptoms were linked to higher psychological and cognitive symptoms. Multivariate analysis found that catastrophizing thoughts were associated with higher anxiety, while positive refocusing was linked to lower anxiety, depression, PTSD, and cognitive symptoms. These findings highlight the role of physical symptoms, psychiatric history, and coping strategies in post-COVID outcomes, underscoring the need for biopsychosocial treatment approaches.

Chen et al. assessed risk and protective factors for sufficient sleep among adolescents during the COVID-19 pandemic using data from the 2021 Adolescent Behaviors and Experiences Survey (*n* = 7,705). Only 23.5% of U.S. high school students reported getting sufficient sleep. Factors as sociated with sufficient sleep included younger age, heterosexual identity, absence of poor mental health, no feelings of sadness or hopelessness, no food insecurity, no emotional abuse, and no schoolwork difficulty. The findings suggest that addressing these factors can help improve sleep duration in adolescents, especially during future pandemics.

Li et al.'s study examined the prevalence and correlates of mental health disorders among Chinese primary healthcare (PHC) physicians and nurses in 2022, post-pandemic. A national sample of 4,246 respondents was surveyed using various mental health assessment tools. Results indicated a decrease in most mental health disorders compared to the early pandemic, except for somatization, phobic anxiety, and obsessive-compulsive disorder. Significant risk factors for mental health issues included female gender, multimorbidity, psychiatric history, quarantine experience, lack of social support, and overtime work. The study highlights the need for regular assessments and psychological support for PHC professionals to address work-related stress and preexisting health conditions.

Wang et al. adapted the Tendency to Stigmatize Epidemic Diseases Scale (TSEDS) into Chinese using the Brislin translation model. They conducted a survey with 434 adults and evaluated various aspects like reliability and validity. The Chinese version of TSEDS consists of 27 items across five dimensions: structural stigma, perceived stigma, organizational stigma, internalized stigma, and social stigma. The scale showed high content validity (0.975), good model fit indices, and strong reliability (Cronbach's alpha = 0.962). Test-retest reliability was 0.912, confirming the scale's consistency and validity.

Nouhi Siahroudi et al. conducted a study to assess the impact of COVID-19 on suicide rates in Iran using an interrupted time series analysis. They analyzed 63,514 suicide cases from April 2009 to March 2023. Results showed a significant increase in the suicide rate during the pandemic, with a 1.003 times increase per month and a 1.1 times rise after accounting for the pandemic. However, no significant interaction between time and COVID-19 was found. While the suicide rate was already rising before the pandemic, a notable increase in suicide deaths occurred 3 months after the pandemic began, suggesting COVID-19 may have influenced suicide rates.

Huang et al.'s study investigated factors affecting the sleep quality of frontline medical personnel during the peak of the COVID-19 pandemic in Shanghai. A cross-sectional survey (June 25–July 14, 2022) analyzed weight change, job title, and tea consumption using PSQI scores. Data from 1,326 participants were split into training (80%) and validation (20%) sets. Six predictive models were tested, with the deep learning (DL) model showing the best predictive performance (AUC = 0.656, specificity = 86.1%, sensitivity = 45.5%). Sleep quality was influenced by multiple factors, with DL emerging as the most effective predictive tool.

In conclusion, the editors wish to thank all the authors, the reviewers, and the editorial board members for contributing to this Research Topic. As was the case during the COVID-19 pandemic, emerging infectious diseases pose a serious threat to public health worldwide and their impact is not only on physical health but also on mental health. We hope that this Research Topic will inspire future novel research approaches in the field of mental health.
